# Homœopathic Medical Society of Pennsylvania

**Published:** 1885-10

**Authors:** 


### HOMŒOPATHIC MEDICAL SOCIETY OF PENNSYLVANIA.

The twenty-first annual session of the Homœopathic Medical Society of Pennsylvania was held in this city September 16th, 1885. Dr. B. F. Betts, President of the Philadelphia Society, made an address of welcome; Dr. John E. James delivered the annual address. Papers on medical and surgical and sanitary matters were read by Dr. C. G. Raue, Dr. A. Korndoerfer, Dr. E Fornais, Dr. Mary Branson, Dr. H. J. Evans, Dr. J. B. Wood, Dr. P. Dudley, Dr. B. W. James, Dr. E. C. Parsons, Dr. J. H. McClelland, Dr. L. H. Willard, Dr. C. M. Thomas, Dr. W. B. Van Lennep, Dr. I. G. Smedley, Dr. C. H. Hofmann. On Thursday the following papers were read: "On Some Fever Experiences," by C. Mohr, M. D.; "Acute Tuberculosis After Measles," by J. C. Morgan, M. D.; "Exophthalmic Goitre," by Joseph E. Jones, M. D.; "A Case of Hysteria with Choreiform Symptoms," by Clarence Bartlett, M. D.; "Clinical Facts," by A. P. Bowie, M. D.; "Clinical Case," by W. J. Martin, M. D.; "Two Clinical Cases," by C. C. Rinehart, M. D.; "Brain Tumors," by A. Thomas, M D.; "A Special Case of Intestinal Obstruction and Fæcal Vomiting," by J. C. Morgan, M. D. In the evening a reception and collation was held in Parlor C, Continental Hotel.

